# Multifocal electroretinography changes at the 1-year follow-up in a cohort of diabetic macular edema patients treated with ranibizumab

**DOI:** 10.1007/s10633-017-9601-2

**Published:** 2017-08-04

**Authors:** Marc Baget-Bernaldiz, Pedro Romero-Aroca, Angel Bautista-Perez, Joaquin Mercado

**Affiliations:** 1 Ophthalmic Service, University Hospital Sant Joan, Reus, Spain; 2 Institut de Investigacio Sanitaria Pere Virgili [IISPV], University Rovira and Virgili, Tarragona, Spain

**Keywords:** Multifocal electroretinogram, Retinal density of response, Implicit time, Diabetic macular edema, Diabetic retinopathy, Ellipsoid zone, External limiting membrane, Central retinal thickness, Total macular volume

## Abstract

**Purpose:**

To determine the changes in the multifocal electroretinogram (mfERG) at 1 year in a clinical series of diabetic macular edema (DME) patients treated with ranibizumab (RNBZ) using a pro re nata protocol.

**Methods:**

We analyzed a clinical series of 35 eyes of 35 patients with DME at baseline and after treating them with RNBZ over 1 year, in order to determine the change in the macular function, which was assessed by means of the response density and the implicit time of the first-order kernel (FOK) P1 wave of the mfERG at the foveola (R1), fovea (R2) and parafovea (R3). These electrophysiological parameters were studied taking into account different independent variables, such as DME type, degree of diabetic retinopathy (DR), level of preservation of both the ellipsoid zone (IS/OS) and the external limiting membrane (ELM) and changes in central retinal thickness (CRT) and total macular volume (TMV). We also studied the relationship between the response density and the best-corrected visual acuity (BCVA).

**Results:**

Eyes with cystic and spongiform DME showed better response density with respect to the serous type (*p* < 0.001) at baseline. Similarly, eyes with high IS/OS and ELM preservation rates showed higher initial response density compared to the others (*p* < 0.001). Eyes with moderate DR had better response density compared to those with severe and proliferative DR (*p* = 0.001). At the beginning of the study, those eyes with proliferative and severe DR showed longer implicit times with respect to those with moderate DR (*p* = 0.04). The response density significantly increased in eyes that anatomically restored the IS/OS and the ELM after being treated with RNBZ (both *p* < 0.001). Similarly, eyes with spongiform DME further improved the response density with respect to those with cystic and serous DME (*p* < 0.001). On the contrary, eyes with hard exudates showed less improvement in their response density at the end of the study (*p* < 0.001). We observed a significant relationship between BCVA and the response density achieved at the end of the study (*p* = 0.012). Eyes with severe and proliferative DR significantly shortened implicit time compared to those with moderate DR (*p* = 0.04).

**Conclusions:**

The multifocal electroretinogram allowed us to differentiate groups of eyes with DME according to their electrophysiological profile, both initially and after being treated with RNBZ. Ranibizumab increased the response density in all DME types included in the study, with a maximum response in those eyes with spongiform type. Once treated with RNBZ, the macular electrophysiological activity improved in eyes that had a well-preserved ellipsoid zone and ELM. The presence of hard exudates was a limitation to the response density achieved at the foveola.

## Introduction

Diabetic macular edema (DME) is the leading cause of visual acuity loss in diabetic patients [1]. Clinical manifestations include the presence of fluid or lipids (hard exudates) in the macula, with an increase in its volume and thickness.

Optical coherence tomography (OCT) is the technique most often used during follow-up for DME and helps to determine the need for continued treatment.

There are different pathophysiological mechanisms involved in the genesis of DME. Several studies have emphasized the role of vascular endothelial growth factor (VEGF) after having observed an increase in its levels in the vitreous of patients with diabetic retinopathy (DR). It increases the retinal vascular permeability that can lead to DME [2]. Currently, the majority of treatments for DME target VEGF to block its action. Ranibizumab (RNBZ) is a selective anti-VEGF drug used in this treatment. Several studies have demonstrated its safety and efficacy in the treatment of this disease [3, 4].

Multifocal electroretinography (mfERG) is a noninvasive test that allows us to assess the functional status of the macula. Patients must fix their eyes on the central part of the monitor, and the macula is stimulated by a sequence of scaled hexagonal flashes of light projected onto it. The matrices most commonly used in clinical practice are 103 and 61 hexagons. In our study, we chose the latter, aiming to shorten the scanning time and thus minimize artifacts due to patient fatigue. The mfERG is taken under conditions of light adaptation in order to obtain an electrophysiological response from the cones [5].

The aim of this study was to evaluate the macular functional status using the mfERG in a population of type 2 diabetic patients with DME over a 1-year follow-up of treatment with RNBZ. We believe that this study will increase our understanding of certain physiological mechanisms involved in the clinical response to RNBZ by determining the electrophysiological response of the macula.

## Methods

### Study design

A prospective clinical study was conducted from January 1, 2014, to December 31, 2014, including a total of 35 eyes of 35 Caucasian patients with DME.

### Ethical adherence

This study was conducted in accordance with local legal requirements (the local ethics committee of *Hospital Universitari Sant Joan de Reus,* approval no. 11-05-26/proj5), and with the revised guidelines of the Declaration of Helsinki. Informed consent was obtained from all participants in the study.

### Power of the study

We estimate the detection of a 95% increase in risk with an accuracy interval of 3%. The calculations were based on the consideration of variables involving two possible random errors in their determination.

1. Response density of the FOK P1 wave: to allow for technical mistakes, a possible error was considered of 20 nanovolts per degree squared (nV/deg^2^) for every ring studied.
2. Error in measuring the thickness of the retina with optical coherence tomography (OCT), with a sampling error of 5 microns, was considered due to possible failure of the technique used.

### Inclusion criteria

Type 2 DM patients with naïve DME diagnosed as clinically significant macular edema with foveal center involvement [6].

### Exclusion criteria

- Patients with type 1 DM
- Patients with cataracts or other opacities
- Patients with uncontrolled glaucoma or previous ocular surgery
- Patients with tractional DME
- Patients with macular ischemia
- Patients with previous nephropathy, stroke or myocardial infarction

### Methods

Diabetic macular edema was diagnosed by a retina specialist in the Ophthalmology service. All examinations were made at baseline, at 6 and at 12 months of follow-up and included BCVA, biomicroscopy, fluorescein angiography (FA), optical coherence tomography (OCT) and multifocal electroretinography (mfERG).

OCT was taken using a TOPCON 3D OCT-2000, and we determined the central retinal thickness (CRT), the total macular volume (TMV), the level of preservation of both the inner segment/outer segment (ellipsoid zone) and the external limiting membrane (ELM) and the presence of hard exudates (HE) in the fovea.

We evaluated quantitatively both the ellipsoid zone and the ELM by measuring the traceable segments for each layer in the fovea. Both layers were classified for each eye as having high, medium or low preservation rate. The level of preservation for each layer was high when it could be measured between 66 and 100% of their length (1000–1500 microns), medium if it was between 33 and 66% (500–1000 microns) and low if it was less than 33% (<500 microns) [7].

We classified the type of DME for each eye according to Otani et al. [8] as having spongiform, cystic or with serous retinal detachment. We also classified the type of DR for each eye according to the International Clinical Diabetic Retinopathy by Wilkinson (2002).

The mfERG was carried out with a RETI-port gamma plus ROLAND^®^ model, according to the recommendations issued by the International Society for Clinical Electrophysiology of Vision (ISCEV) in 2011 [9]. Technique: the patient was placed in front of an LCD 19 monitor, onto which we projected a hexagonal matrix of 61 flicker lights, transitioning from white to dark in high frequency (75 Hz); this pattern of stimulation was presented under photopic conditions to achieve electrophysiological responses of the cone and bipolar cells. The correct fixation of each tested eye was monitored by external observation.

The basic mfERG response includes a first negative wave (N1) followed by a positive wave (P1). The set of N1–P1 biphasic waveforms is known as the first-order kernel (FOK) or first-order response [10].

The two electrophysiological variables selected in our study to assess the electrophysiological responses at the foveola, fovea and parafovea were the response density and the implicit time of the P1 wave of the first-order kernel (FOK). The first ring (R1) corresponds to 0° to 2° of the visual field (foveola), the second ring (R2) corresponds to 2° to 5° of the visual field (fovea), and the third (R3) corresponds to 5° to 10° of the visual field (parafovea).

The response density is the amplitude achieved per unit of area measured in nanovolts per square degree (nV/deg^2^). It quantifies the amplitude obtained in each ring, considering its size. It is at its maximum at the foveola since that is the macular region with the highest cone density, and decreases with the eccentricity because the cone density is reduced by the same proportion. The implicit time is the time it takes to reach the maximum amplitude in every macular region studied.

These three rings are affected by DME and are responsible for the changes in BCVA.

Normal electrophysiological values in the present study are:

- Response density:
  1. 60–80 nV/deg^2^ in R1
  2. 40–60 nV/deg^2^ in R2
  3. 20–40 nV/deg^2^ in R3
- Implicit time:
  1. 41–43 ms in R1
  2. 39–41 ms in R2
  3. 37–39 ms in R3

To obtain the normal electrophysiological values in our population, we studied thirty healthy volunteers from our hospital, fifteen participants aged between 50 and 60 and fifteen between 60 and 65.

Ranibizumab (RNBZ) was injected at doses of 0.5 mg in 0.05 ml, according to the pro re nata (PRN) protocol, with injections at baseline and at 1-month and 2-months, a total of three injections as a loading dose administered to all patients. Next, we evaluated the patients and determined the follow-up injections or next control according to the presence of fluid in the macula (more than 300 microns) and/or if BCVA was reduced by 5 letters.

### Statistical methods

The data were analyzed using the SPSS software package, version 22.0. In this study, the dependent variables were the response density of the P1 wave, the implicit time of the P1 wave of the FOK of the mfERG measured in the three first rings, and the BCVA. The independent variables were age, sex, DM duration, HbA1c level, type of DME, central retinal thickness (CRT) and total macular volume (TMV) measured by optical coherence tomography (OCT) and the level of preservation of both the ellipsoid zone and the ELM.

The normal data curve was evaluated using the Kolmogorov–Smirnov test. A descriptive statistical analysis of the quantitative data was made. For qualitative data, we used the analysis of frequency and percentage in each category. Differences between variables included in the analyses were examined using Student’s *t* test, to compare two variables, or one-way ANOVA, to compare more than two variables. Inferential analysis for qualitative data was carried out using the Chi-square table and the determination of McNemar’s test for categorical data or Cochran’s *Q* test when there were more than two fields. For continuous quantitative data, we used Pearson’s parametric coefficient and Spearman’s coefficient for qualitative parametric variables. Cox regression survival analysis was applied in the study of BCVA and the response density of the P1 wave in the foveola (R1) as dependent variables.

## Results

Table 1 reflects the demographic data of the patient sample. A total of 35 eyes of 35 patients with DME were studied; 57.15% of the patients were women, all patients had type 2 diabetes mellitus, 68.57% were treated with insulin, and the mean HbA1c was 7.9 ± 1.07% (6.3–10). Nobody presented with mild diabetic retinopathy, and severe and proliferative diabetic retinopathy affected 40% of the patients.

**Table 1:** Demographic data of the patients in the study

Variable	Value
Mean age	54 ± 6.2 years (53–77)
Sex	20 women (57.15%)
	15 men (42.85%)
Arterial hypertension	28 patients (80%)
Mean DM duration	21.3 ± 10.7 years (4–40)
HbA1c mean	7.9 ± 1.07% (6.3–10)
DM treatment	
Insulin	24 patients (68.57%)
Oral drugs	11 patients (31.43%)
Diabetic retinopathy level	
Moderate DR	21 patients (60%)
Severe DR	7 patients (20%)
Proliferative DR	7 patients (20%)
Hard exudates in the center of the fovea	16 patients (45.71%)
Preservation of the ellipsoid zone	
≥66% of its length at the fovea (≥1000 μm)	14 patients (40%)
≥33% but ≤66% (from 500 to 1000 μm)	11 patients (31.42%)
≤33% (≤500 μm)	10 patients (28.58%)
Preservation of the ELM layer	
≥66% of its length at the fovea (≥1000 μm)	13 patients (37.14%)
≥33% but ≤66% (from 500 to 1000 μm)	10 patients (28.58%)
≤33% (≤500 μm)	12 patients (34.28%)

The characteristics of DME are presented in Tables 1 and 2. The mean initial CRT was 446.1 ± 121.3 μm (282–708), with a total macular volume (TMV) of 9.93 ± 1.56 mm^3^ (6.68–13.9). Hard exudates affecting the center of the fovea were observed in 45.71% of eyes.

**Table 2:** Mean value changes in the study

	Baseline	6 months	12 months	Significant changes
BCVA	46.7 ± 16.47 ETDRS letters (20–73)	60.3 ± 13.7 ETDRS letters (20–82)	59.1 ± 17.2 ETDRS letters (20–80)	*p* < 0.001, *F* = 4.168
CRT	446.1 ± 121.3 μm (282–708)	389 ± 139.7 μ (130–715)	346 ± 132.8 μ (139–653)	*p* = 0.001, *t* = −4.351
TMV	9.93 ± 1.56 mm3 (6.68–13.9)	9.42 ± 1.6 mm3 (6.85–13.18)	9.12 ± 14.56 mm3 (6.67–14.56)	*p* = 0.003, *t* = −3.694
Mean preservation of the ellipsoid zone	826.8 ± 450.5 μm	917.79 ± 451.90 μm	935.64 ± 467,9 μm	*p* = 0.009, *F* = 2.766
Mean preservation of the ELM layer	662.7 ± 498,2 μm	712.81 ± 432.2 μm	763.64 ± 517,78 μm	*p* = 0.002, *F* = 3.981
Mean dR P1 wave in R1	30.6 ± 15.4 nV/deg^2^ (9.5–63.7)	40.5 ± 22.3 nV/deg^2^ (9.8–93.7)	47.9 ± 24.9 nV/deg^2^ (5.6–100)	*p* < 0.001, *F* = 4.528
Mean dR P1 wave in R2	18.7 ± 6.4 nV/deg^2^ (7.6–34.4)	30.5 ± 5.3 nV/deg^2^ (7.6–39)	25.9 ± 3.7 nV/deg^2^ (1.4–35.9)	*p* = 0.05, *F* = −1.137
Mean dR P1 wave in R3	15.3 ± 7.8 nV/deg^2^ (0.8–44.5)	17.2 ± 10.7 nV/deg^2^ (5.54–47.9)	15.4 ± 13 nV/deg^2^ (4.32–79.8)	*p* = 0.91, *F* = −0.063
Mean IT-P1 R1	44.07 ± 7.3 ms (33.6–50.2)	42.2 ± 9.1 ms (19.7–50.2)	41.9 ± 8.9 ms (16.8–50.2)	*p* = 0.26, *F* = 1.128
Mean IT-P1 R2	46.4 ± 3.8 ms (32.5–50.2).	45.5 ± 7.1 ms (19.7–67.4)	44.5 ± 10.2 ms (19.7–79.8)	*p* = 0.32, *F* = 0.948
Mean IT-P1 R3	44.3 ± 5.1 ms (20.1– 50.2)	43.29 ± 5.16 ms (20.2–50.2)	43.86 ± 5.25 ms (23.6–50.2)	*p* = 0.74, *F* = 0.353

*BCVA* best-corrected visual acuity, *CRT* central retinal thickness, *TMV* total macular volume, *ellipsoid zone* internal segment/external segment layer, *ELM* external limiting membrane, *F* ANOVA function

**Fig. 1:**
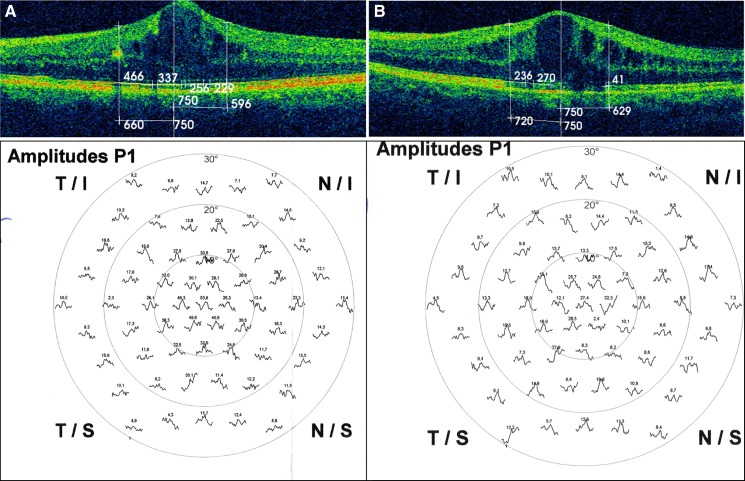
**a** Eye number 5 of the study showed an ellipsoid zone preservation rate of 85%, and its response density of the P1 wave in R1 was 53.8 nV/deg^2^ (89.6% of the normal value), and in R2 was 31.7 nV/deg^2^ (79.2% of the normal value). **b** Eye number 25 had a wide ellipsoid zone disruption (preservation rate of 36.4%), and the response density of the P1 wave in R1 was 27.4 nV/deg^2^ (45.6% of the normal value), and in R2 was 17.3 nV/deg^2^ (43.2% of the normal value)

The baseline preservation of the ellipsoid zone and the ELM are described in Table 1; statistical analysis found a positive relationship between the level of preservation of both layers and the response density achieved (*t* = 0.864, *p* < 0.001), as shown in Fig. 1 corresponding to eyes numbers 5 and 25 of our study.

Table 2 shows the visual function of the studied eyes. The baseline mean best-corrected visual acuity was 46.7 ± 16.47 ETDRS letters (20–73). The values of the response density of the P1 wave at baseline for the three studied rings are also described in Table 2.

### Study of the response density of the P1 wave and its implicit time at baseline

Table 2 also shows the electrophysiological parameters studied at baseline and the changes that appeared at 6 and at 12 months during the treatment with RNBZ. The mean response density of the P1 wave in R1 (foveola) at baseline was 30.6 ± 15.4 nV/deg^2^, compared to a normal value between 60 and 80 nV/deg^2^. Similarly, the initial response density of P1 wave in R2 (fovea) was 18.7 ± 6.4 nV/deg^2^, while the normal range is between 40 and 60 nV/deg^2^. Finally, the mean initial response density of the P1 wave in R3 (parafovea) was 15.3 ± 7.8 nV/deg^2^, with its normal value being between 20 and 40 nV/deg^2^. Thus, a reduction in the initial response density was observed equally at the foveola, fovea and parafovea, with 50% reductions to normal values in the foveola and fovea and a 25% of reduction in the parafovea.

The only independent variable that demonstrated a relationship with the implicit time at baseline was the degree of DR *r* = 0.35 (*p* = 0,04). Eyes with moderate DR had a shorter implicit time than eyes with severe and proliferative DR.

### Study of changes observed at follow-up

**Fig. 2:**
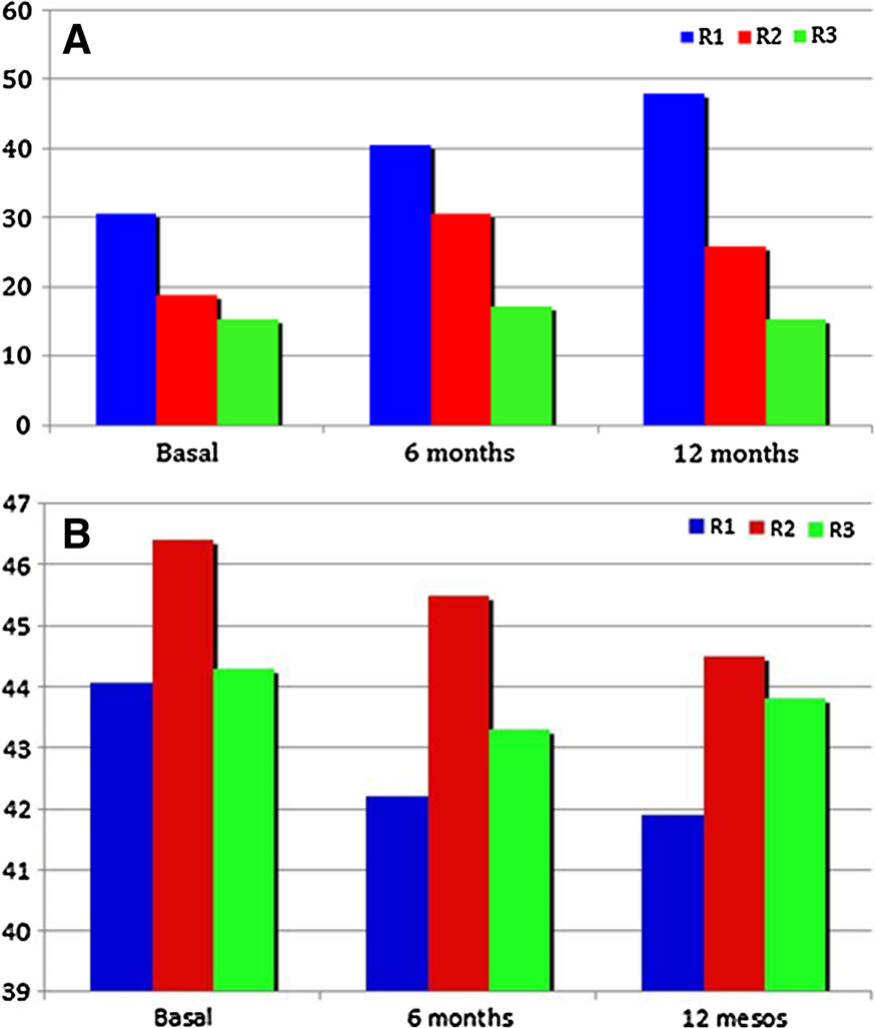
**a** Changes observed in the response density of the P1 wave during the study in the foveola (R1), fovea (R2) and parafovea (R3); Note that the response density increased especially in R1 after the treatment with ranibizumab. **b** Changes observed in the implicit time of the P1 wave during the study in the foveola (R1), fovea (R2) and parafovea (R3). The implicit time decreased in all three rings after the treatment with ranibizumab, but the changes were not significant

Figure 2 and Table 2 show the response density and the implicit time changes of the P1 wave from baseline. The response density increased progressively in R1. However, this parameter showed a slight decrease in R2 and R3 rings at 12 months.

Regarding the severity of diabetic retinopathy (DR), the majority of eyes increased the response density in the foveola at 6 and at 12 months. However, eyes with moderate DR achieved higher response density than those with severe and proliferative DR (Student’s *t* test: *t* = −10.441, *p* < 0.001). On the other hand, eyes that shortened the implicit time most were those with severe and proliferative DR (*r* = 0,38; *p* = 0.035).

**Fig. 3:**
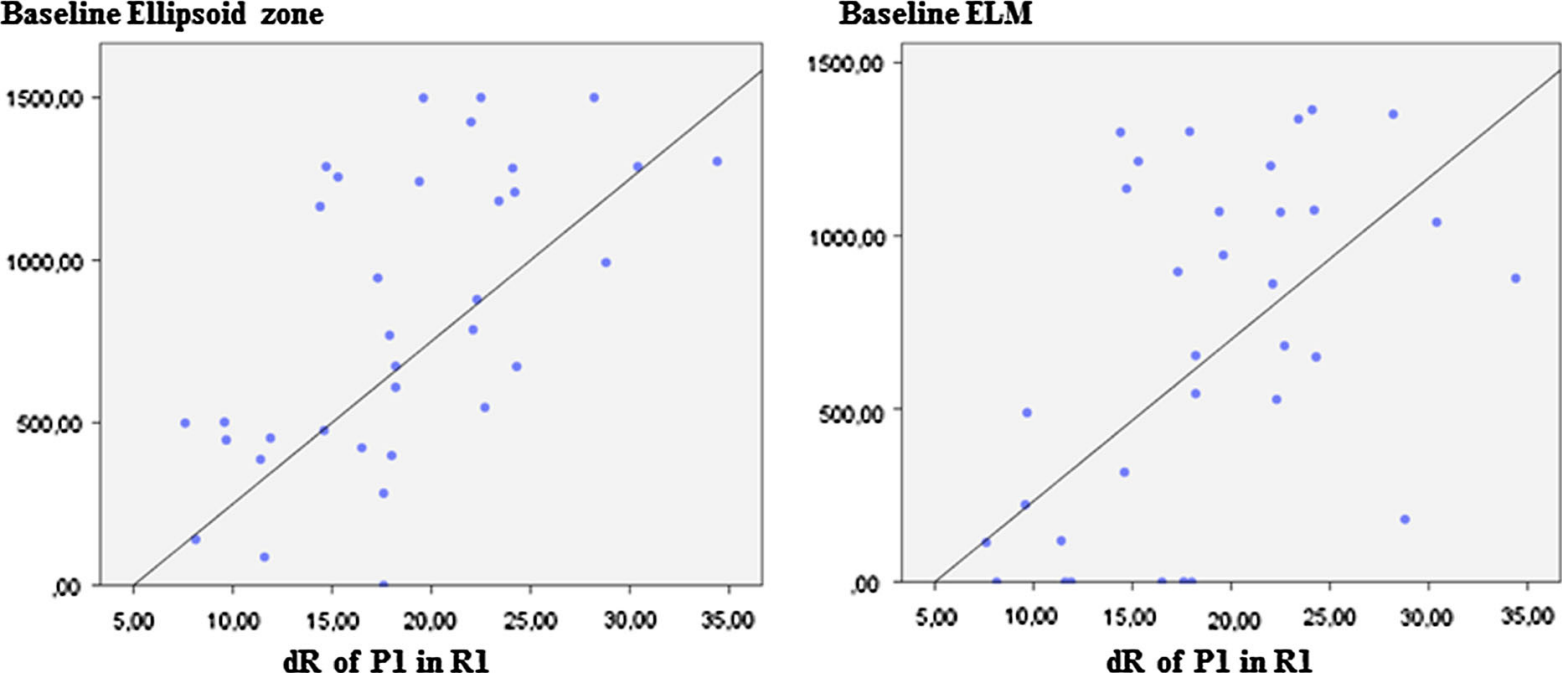
Scatter plot of the relationship between the response density of the P1 wave in the foveola (R1) compared to the preservation of the ellipsoid zone and the ELM, before treating with ranibizumab. The greater the level of preservation of both layers at the beginning of the study, the higher the response density achieved in the foveola

In the statistical study, the initial response density of the P1 wave in the foveola showed a significant relationship with the level of preservation of the ellipsoid zone and the ELM at the beginning of the study (Fig. 3):

1. Ellipsoid zone results:
  The mean ellipsoid layer definition had increased at the 1-year follow-up from 826.8 ± 450.5 μm to 935.64 ± 467.9 μm; the differences were significant according to ANOVA testing, with *F* = 3.981 and *p* = 0.002.
  The baseline level of preservation of the ellipsoid zone correlated significantly with the final response density of the P1 in R1 (*r* = 0.84, *p* < 0.001) and R2, (*r* = 0.62, *p* = 0.04) but not in R3 (*r* = 0.14, *p* = 0.83). Eyes that partially or totally recovered the ellipsoid zone increased the response density of the P1 wave with significant values in R1 (*r* = 0.82, *p* < 0.0001) but not in R2 or R3.
2. ELM results:
  The mean ELM layer definition had increased at the 1-year follow-up from 662.7 ± 498.2 μm to 763.64 ± 517.78 μm; the differences were significant according to ANOVA testing, with *F* = 3.981 and *p* = 0.002.
  The baseline preservation of the ELM correlated significantly with the final response density of the P1 wave in R1 (*r* = 0.83, *p* < 0.001) and in R2 (*r* = 0.62, *p* = 0.003) but not in R3 (*r* = 0.13, *p* = 0.46). Eyes that showed a recovery of the ELM increased their response density of the P1 wave with significant values at only the foveola (*r* = 0.81, *p* < 0.0001), as shown in Fig. 6 in case number 33 of our study.

Table 2 and Fig. 7 show that during this study the mean CRT and TMV decreased. In the statistical study, we observed a significant relationship between the decrease in both the CRT and the TMV and the increase in the response density in R1. The CRT decrease at the end of the study was significant by Students’ *t* test , with values of *t* = 0.75 and *p* = 0.03, and the TMV statistical study showed values of *t* = 0.61 and *p* = 0.04.

**Fig. 4:**
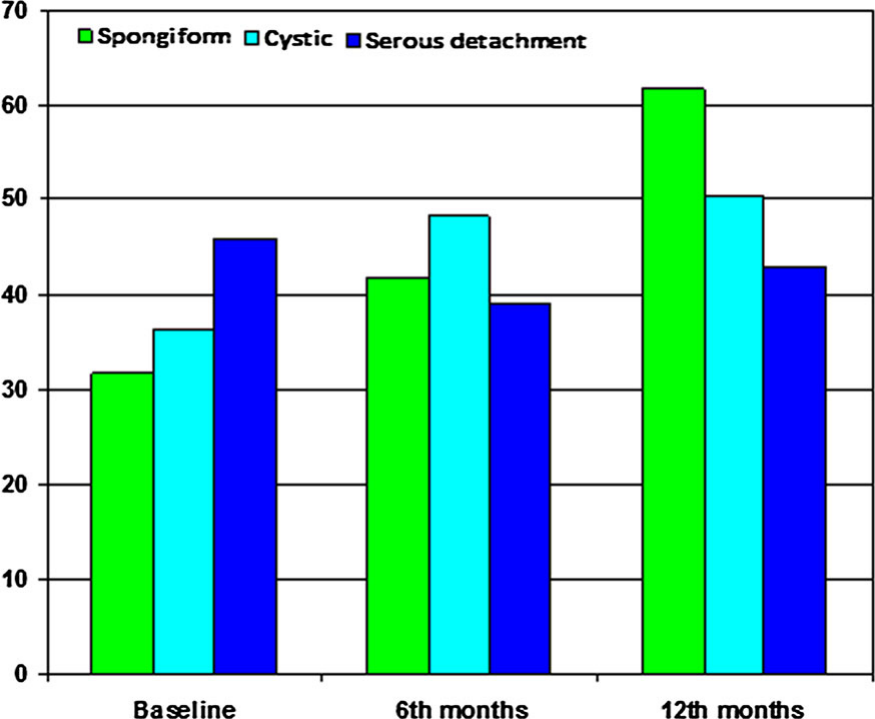
Relationship between the response density of the P1 wave in the foveola (R1) measured in nV/deg^2^ at the beginning of the study, at 6 and at 12 months in the three types of DME. The eyes with the spongiform type further improved the response density after being treated with ranibizumab

According to DME type, spongiform improved the response density more than the serous and cystic types at 6 months (*r* = 0.58, *p* = 0.013) and at 12 months (*r* = 0.77, *p* < 0.0001) (Fig. 4).

The initial presence of hard exudates (HE) in the center of the fovea correlated with low levels of response density in R1 (*r* = 0.71, *p* = 0.02). The mean response density increased by 21.94 ± 6.452 nV/deg^2^ in patients without HE and by 13.25 ± 4.363 nV/deg^2^ in patients with HE in R1, and the differences were significant by Student’s *t* test (*p* = 0.001).

The number of injections did not correlate with the response density achieved in the macula (*p* = 0.72). The majority of eyes received 6 injections (43%). Other variables, such as age, sex, DM duration and HbA1c levels, were not significant with the electrophysiological parameters studied.

**Fig. 5:**
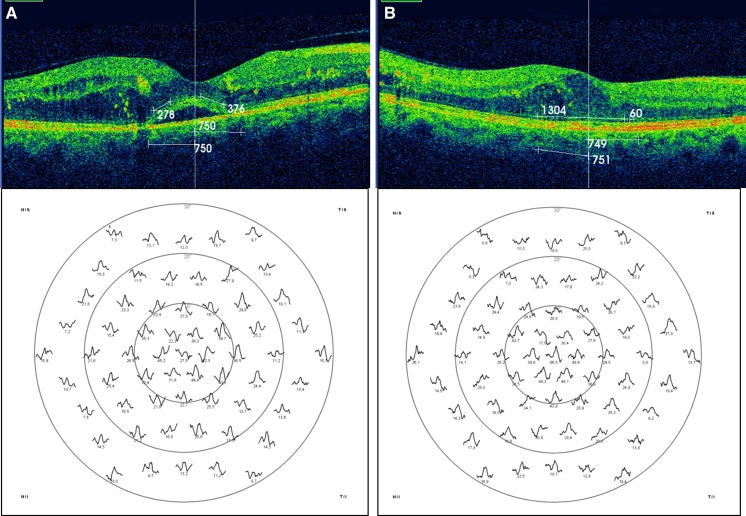
**a** Eye number 31 of the study had initial serous DME type with an ELM preservation rate of 43%, and the response density of the P1 wave in R1 was 27.5 nV/deg2 (46% of the normal value). **b** Eye number 31 of the study showed a recovery of the ELM layer after being treated with ranibizumab (preservation rate of 91%), and the response density of the P1 wave achieved in R1 after being treated with ranibizumab was 66.5 nV/deg^2^ (normal value)

Baseline BCVA was 46.7 ± 16.47 ETDRS letters (20–73). At 6 months of follow-up it was 60.3 ± 13.7 ETDRS letters (20–82) and at the final 1-year follow-up it was 59.1 ± 17.2 ETDRS letters (20–80), with changes significant in the statistical study (*p* < 0.001). The mean change in BCVA at 6 months was 13.58 ± 11.22 letters, and at 12 months, it was 12.41 ± 14.01 letters. The changes show that eyes with the best visual acuity recovery at 6 months were also those with the best final visual acuity (Fig. 5).

**Fig. 6:**
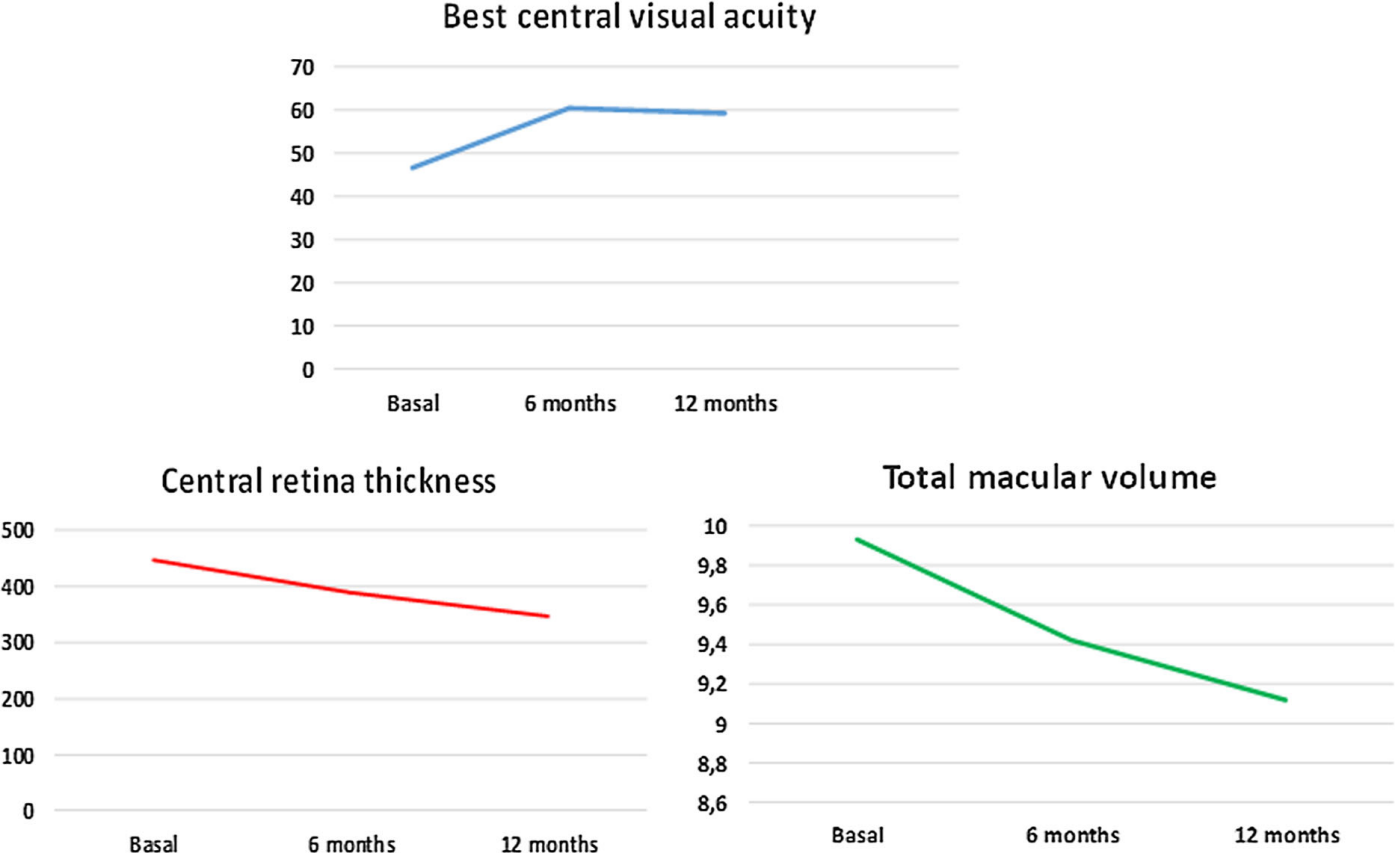
**a** Mean best-corrected visual acuity changes during the study measured in ETDRS letters. Eyes improved significantly the visual acuity at 6 months and then, remained stable. **b** Mean, central retinal thickness changes during the study. Eyes reduced significantly the CRT after being treated with ranibizumab. **c** Total macular volume changes during the study. Eyes reduced significantly the TMV at the end of the study

Our study demonstrates an increase in BCVA in eyes with a decrease in CRT (*p* = 0.02) and TMV (*p* = 0.01) (Table 2; Fig. 6). The study of the preservation of the ellipsoid zone and the ELM layers and BCVA yielded values of *p* < 0.001 for the ellipsoid zone and *p* < 0.001 for the ELM layer. Therefore, eyes with better initial preservation of both layers, further improved the visual acuity. By contrast, eyes with the presence of HE in the macula (*p* < 0.001) or those diagnosed with more severe DR (*p* = 0.01) showed less recovery of the BCVA (*p* = 0.01). Other variables such as age, sex, DM duration, DM treatment, HbA1c level or the number of injections administered did not correlate with the final BCVA.

**Fig. 7:**
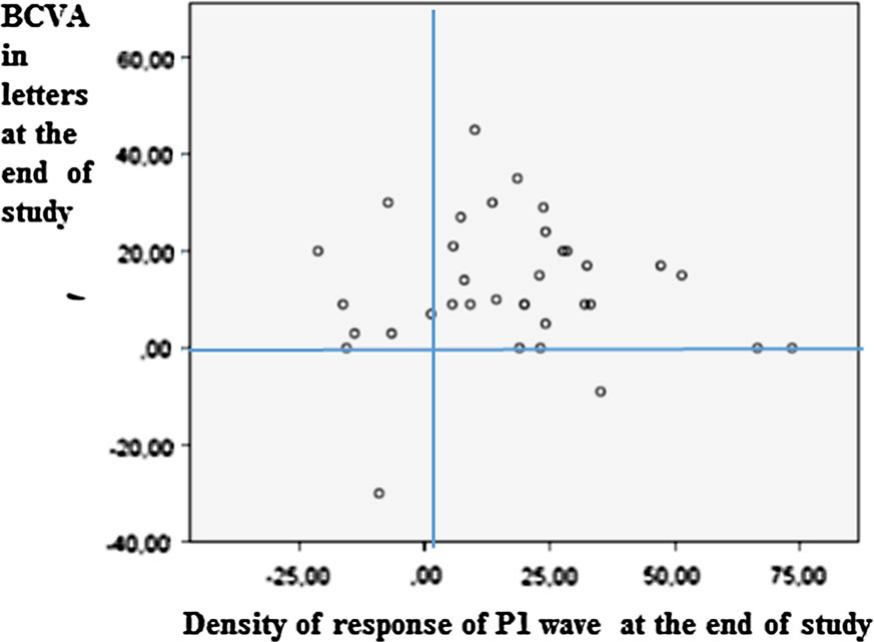
Scatter plot of the relationship between BCVA and the response density of the P1 wave in R1. Eyes that demonstrated better BCVA at the end of the study were those with higher response density in R1

Changes in BCVA and the response density of P1 wave in R1 at the end of the study are shown in the Fig. 7. The statistical study of changes at the 1-year follow-up between the BCVA and the response density of the P1 wave in R1 was significant *p* = 0.012 after adjusting the data for age and sex.

### RNBZ injection study

In this study of DME treatment, the mean number of injections of RNBZ was 5.5 ± 0.9 (3–7). Most patients received five (28.6% of patients) or six injections (42.9% of patients), with no adverse effects observed. We did not find any relationship between the number of injections and the increase in the response density of the P1 wave or the implicit time in the first three rings at 6 or 12 months.

## Discussion

The multifocal electroretinogram can also be used to study the functional status of the macula divided into rings in an objective manner. It is therefore very useful for monitoring diseases affecting the macular area, such as diabetic macular edema.

Current reports on retinal electrophysiology usually present data from the response density and the implicit time of the P1 wave in the five rings from the center of the retina. Our study focused on evaluating these two electrophysiological parameters in the first three rings, because they are commonly affected in cases of DME with center involvement. Changes observed in the first 10° of the central visual field, corresponding to sum of R1–R3, are responsible for changes in BCVA.

Notably, the LUCIDATE study [11], which compared RNBZ and laser, is the only published report on RNBZ treatment and multifocal ERG (mfERG) responses. Other studies have either assessed the mfERG changes after the injection of a dexamethasone implant [12] or triamcinolone acetonide injections, which have shown no significant changes at the 6-month follow-up [13].

Our results demonstrate low levels of the response density in eyes with DME at baseline in the first three rings, at 50% of the normal value. These levels were higher than reported in the LUCIDATE study [11], which found a 70% reduction in 33 eyes with DME. The different inclusion criteria of our study might explain these differences, although both studies found a functional alteration in patients with DME.

As in other studies, we observed a positive relationship between altered ellipsoid zone and ELM layers and BCVA changes [14–17]. We also observed a positive relationship between the response density and the preservation of the ellipsoid zone and ELM layers in the fovea (R1–R2). We can therefore suppose that the values obtained in the response density in the fovea resulted from the anatomical and functional status of the outer retinal layers. The generation of the biphasic wave of the first-order response of the mfERG is known to be due to the electrical activity of the ON–OFF bipolar cells through the actions of the cones [18]. Hence, dysfunction or density loss of cones in the hexagon ring (loss of preservation in the ellipsoid zone and ELM) indicates that there is a critical density of bipolar cells that cannot be stimulated. As a result, there is a reduction in the response density of the P1 wave in the corresponding ring. Since the fovea contains the highest density of cones, we believe that an alteration of the ellipsoid zone affecting this macular region might generate a greater reduction in the response density with respect to other regions of the macula.

Regarding the type of DME, spongiform eyes improved the response density in the fovea more than cystic and serous types did. A possible explanation for that is that liquid vacuoles in spongiform eyes are smaller than in the other two forms and they might interfere less with the cones and bipolar cell functions. It has been postulated that big intra-retinal cysts might result in cone dysfunction by toxic and anatomical interference [19]. Furthermore, a much greater dysfunction of both blood retinal barriers in the serous type has been demonstrated [20]. Eyes that presented with hard exudates in the fovea at the beginning of the study signaled a poor prognosis with a smaller improvement in the response density. Several mechanisms might be involved, for example lipids might interfere both with the arrival of the stimulus at the photoreceptors and at the same time, block the response they generate to the corneal electrodes. We should also bear in mind that hard exudates induce local inflammation by destroying retinal cells around them. If the fovea is affected, there is a decrease in retinal function along with a decrease in the magnitude of the response density on the mfERG.

The severity of diabetic retinopathy correlated with both the implicit time and the response density and both at the beginning and at the end of the study. The higher the degree of DR, the lower the response density and the longer the implicit time in the fovea, as other studies have previously reported [21, 22]. Eyes with more severe DR suffer from chronic retinal inflammation and finally apoptosis.

The study of other variables, such as age, sex, DM duration, insulin treatment and HbA1c levels, was unrelated to the electrophysiological profile in the three central rings. Furthermore, these metabolic and demographic variables did not influence the amelioration of electrophysiological status after a 1-year follow-up. Similarly, the RISE and RIDE studies [23] reported similar results, demonstrating that BCVA had no relationship with glycaemic control, blood chemistry or renal function.

In our study, BCVA and the response density in the fovea correlated. Therefore, we can assume that both variables are useful for measuring the functionality of the central retina and for monitoring those eyes with diabetic macular edema being treated with ranibizumab. The strength of the study is that, as far as we know, this is the first to report on a homogeneous sample of DM type 2 patients with DME who have been monitored in parallel with the electrophysiological profile, as well as the anatomical status of the ellipsoid zone and ELM layers after treatment with ranibizumab. Limitations include the sample size, the PRN technique and the length of the 1-year follow-up.

In conclusion, multifocal electroretinogram seems to be an effective technique for objectively monitoring eyes with DME treated with ranibizumab. Eyes that further improved the electrophysiological profile were those with high level of preservation of the ellipsoid zone and ELM, moderate DR and no presence of hard exudates. Ranibizumab improved the electrophysiological profile in eyes with DME, mainly in those with the spongiform type.
